# Pterostilbene inhibits inflammation and ROS production in chondrocytes by activating Nrf2 pathway

**DOI:** 10.18632/oncotarget.16716

**Published:** 2017-03-30

**Authors:** En-Xing Xue, Jian-Ping Lin, Yu Zhang, Sun-Ren Sheng, Hai-Xiao Liu, Yu-Long Zhou, Hui Xu

**Affiliations:** ^1^ Department of Orthopedic Surgery, The Second Affiliated Hospital and Yuying Children's Hospital of Wenzhou Medical University, Wenzhou, Zhejiang, 325027, China; ^2^ Department of Orthopedic Surgery, Hainan Provincial People's Hospital, Haikou, Hainan, 570311, China

**Keywords:** pterostilbene, nuclear factor erythroid 2-related factor 2, chondrocyte, inflammation, reactive oxygen species

## Abstract

Pterostilbene has been reported as a potential drug to inhibit oxidative stress and inflammation. However, the effect of pterostilbene on chondrocytes and osteoarthritis remains to be elucidated. We sought to investigate whether pterostilbene could protect chondrocytes from inflammation and ROS production through factor erythroid 2-related factor 2 (Nrf2) activation. The pterostilbene toxicity on chondrocytes collected from cartilages of Sprague-Dawley rats was assessed by CCK-8 test. Immunofluorescence and Western blotting explored the nuclear translocation of Nrf2. Nrf2 expression was silenced by siRNA to evaluate the involvement of Nrf2 in the effect of pterostilbene on chondrocytes. Finally, osteoarthritis model was established by the transection of anterior cruciate ligament and partial medial meniscectomy in rats, and then these rats received pterostilbene 30 mg/kg, daily, p.o. for 8 weeks. Histology and immunohistochemistry were used to assess histopathological change and Nrf2 expression in cartilage. Nuclear translocation of Nrf2 was stimulated by pterostilbene without cellular toxicity. Pterostilbene inhibited the level of COX-2, iNOS, PGE2, and NO, as well as the mitochondrial and total intracellular ROS production induced by IL-1β in chondrocytes, partially reversed by the Nrf2 silencing. Pterostilbene prevented cartilage degeneration and promoted the nuclear translocation of Nrf2 in cartilage. These results suggest that pterostilbene could inhibit the IL-1β-induced inflammation and ROS production in chondrocytes by stimulating the nuclear translocation of Nrf2.

## INTRODUCTION

Osteoarthritis (OA), a most common joint disease, causes the joint deformity and pain, contributing to work disability and sick leave [1]. The erosion and destruction of articular cartilage are the specific characteristics of OA, accompanied by the loss of chondrocyte number and function, as well as the degradation of extracellular matrix (ECM). Recently, the focus on the mechanism and therapy of OA has been shifted from structural to the molecular changes such as inflammation and oxidative stress [2].

Several compelling pieces of evidences have demonstrated the inflammation-associated changes in the OA cartilage [3, 4]. Inflammatory mediators including chemokines, adipokines, and pro-inflammation cytokines such as tumor necrosis factor-alpha (TNF-α), interleukin-1β (IL-1β), IL-4, and IL-6 were increased in the serum and OA cartilage [5, 6]. These cytokines stimulated chondrocytes to secrete synthesize cyclooxygenase 2 (COX-2), nitric oxide (NO), prostaglandin E2 (PGE2), and matrix metalloproteinase (MMPs), leading to the destruction of cartilages [7]. On the other hand, oxidative stress has been regarded as another important etiology for OA, which regulates the intracellular signaling processes, chondrocyte senescence, apoptosis and autophagy, ECM synthesis and degradation [2]. Importantly, reactive oxygen species (ROS) production results in the proinflammatory phenotypic alteration via the nuclear factor-kappa B (NF-ĸB) pathway in OA chondrocytes [8, 9]. The well-acknowledged stimulator of inflammation and ROS production [10], IL-1β, was used to mimic the condition in the OA chondrocytes in the present study.

The main drugs against inflammation and pain in OA are the non-steroidal anti-inflammatory drugs (NSAIDs) with some side effects and high economic burden [11]. However, a reliable and efficient drug for inhibiting ROS remains to be explored. Pterostilbene (PTE), a natural component of blueberry, is a phytoalexin and an analog of resveratrol with higher bioavailability and convenience [12, 13]. Some studies have reported the suppressive effects of PTE on inflammation, oxidative stress, and apoptosis in mammalian cells [14]. Under hyperosmotic stress, PTE reduced the ROS production and the expression of inflammatory mediators including IL-1β, IL-6, TNF-α, metalloproteinase-2 (MMP-2), and MMP-9 in corneal epithelial cells [15]. The oxidative stress induced by low-density lipoprotein in vascular endothelial cells was also inhibited by PTE [16]. In addition, the protective roles of PTE on anti-hyperlipidemia and carcinogenesis have also been reported [17, 18]. However, the role of PTE in chondrocytes and OA has never been investigated. Therefore, we hypothesized that PTE could attenuate the IL-1β-induced inflammation and ROS production.

Nuclear factor erythroid 2-related factor2 (Nrf2) is a basic leucine zipper transcription factor in an intracellular adaptive mechanism against the hostile stimulations of oxidative stress by managing the level of phase II detoxifying enzymes andproteins [19]. Upon stimulation by oxidative stress, the cytoplasmic Nrf2 enters into the nucleus and promotes the transcription of antioxidant response element (ARE)-dependent gene including the heme oxygenase 1 (HO1), NAD(P)H-quinone oxidoreductase-1 (NQO1), glutathione peroxidase (GPx), -glutamylcysteine synthetase (γ-GCS), catalase (CAT), and superoxide dismutase (SOD) [20]. The protective roles of Nrf2 in brain, heart, and liver have been widely reported [21, 22]. Recently, the involvement of Nrf2 in the protective role of PTE on the cell apoptosis and oxidative stress has also been demonstrated [23]. However, the association between Nrf2 and PTE has never been reported in chondrocytes and OA. In the present study, we investigated whether PTE could activate Nrf2 signaling and inhibit the IL-1β-induced inflammation and ROS production, as well as the effects *in vivo*. The OA model of SD rats were used in the present study to investigate the role of PTE on the cartilage degeneration and the nuclear translocation of Nrf2 in cartilages.

## RESULTS

### Effect of PTE on cell viability and nuclear translocation of Nrf2 in the primary chondrocytes

**Figure 1:**
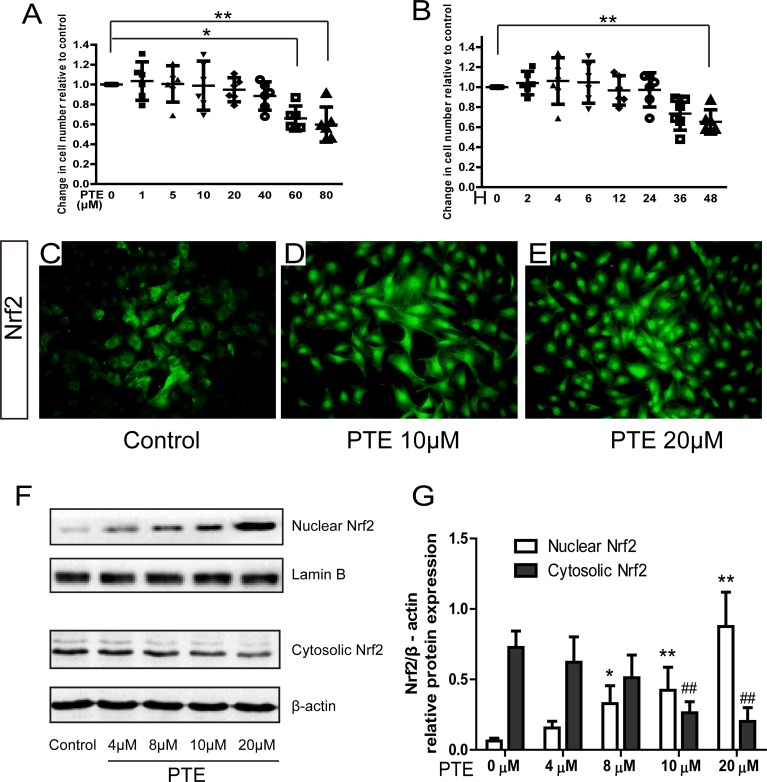
Effect of PTE on cell viability and nuclear translocation of Nrf2 in chondrocytes (**A**–**B**) The effect of PTE on cell viability with different doses and treatment time. (**C**–**E**) The representative images of Nrf2 immunofluorescence in chondrocytes with 10 and 20 μM PTE treatment for 24 h. (**F**) Representative Western blotting bands for nuclear-cytoplasmic distribution of Nrf2 protein treated with 4–20 μM PTE for 24 h. (**G**) The semi-quantitative analysis for the optical density of Nrf2 bands. Data were shown as mean ± 95% CI (standard deviation), *P < 0.05, **P < 0.01 compared with 0 μM group, ^##^*P* < 0.01 compared with 0 μM group, *n* = 6.

Cell viability after PTE treatment was first analyzed in our study. After 24 h treatment, only the chondrocytes treated with 60 μM and 80 μM PTE showed decreased viability (Figure 1A, *P <* 0.05). A significant difference was not observed between 0–40 μM PTE-treated and control chondrocytes. 20 μM PTE treatment for 0–36 hours did not decrease cell viability (Figure 1B, *P <* 0.05), suggesting that the PTE treatment for less than 48 h was not detrimental for rat chondrocytes. Therefore, 20 μM PTE treatment for 24 h was used in the following experiments.

The activation of Nrf2 pathway in chondrocytes was detected by immunofluorescence and Western blotting. In the control chondrocytes, the green fluorescence was mainly found in the cytoplasm. However, the 10 and 20 μM PTE markedly increased the intensity of green staining in the nuclei (Figure 1C–1E), indicating the formation of nuclear translocation. Western blotting detected a similar finding. A dose-dependent change of the nuclear-cytoplasmic distribution of Nrf2 protein was found in the PTE-treated chondrocytes with a significant difference in the 4, 10 and 20 μM PTE-treated cells compared with the control cells (Figure 1F–1G), accompanied by the decrease of cytoplasm protein and increase of the nuclear protein. These results indicated the nuclear translocation of Nrf2 activated by the PTE.

### The involvement of Nrf2 in the anti-inflammation effect of PTE on IL-1β-treated chondrocytes

**Figure 2:**
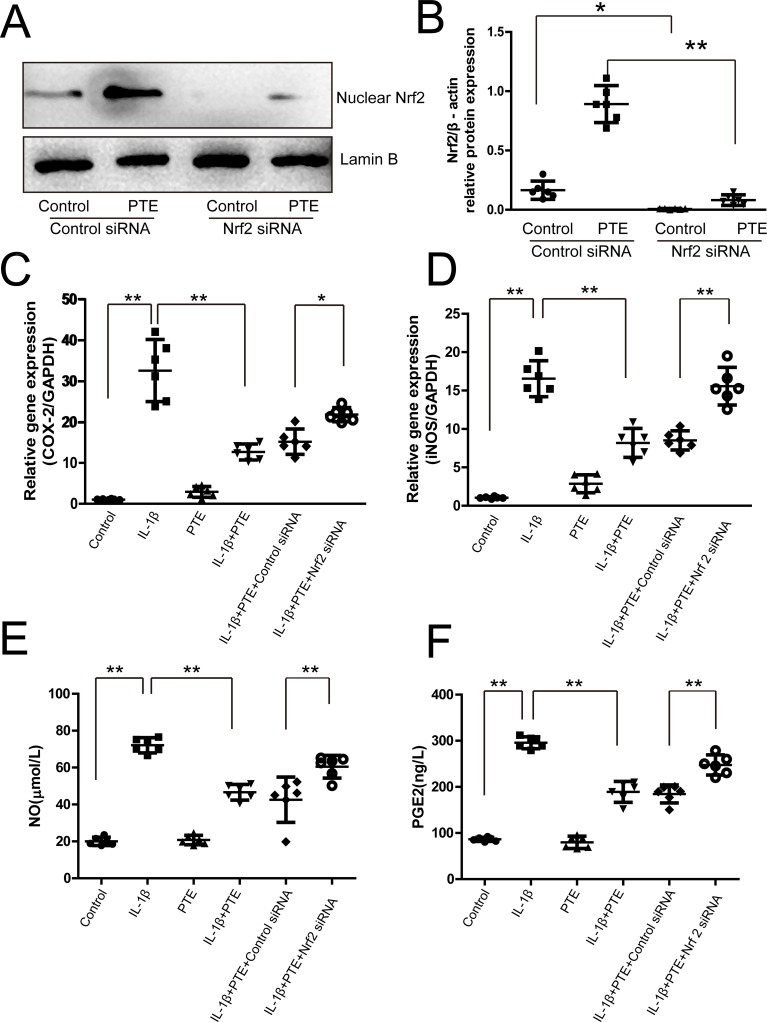
The involvement of Nrf2 in the inhibitory effect of PTE on the expression of inflammatory mediators in the chondrocytes treated with IL-1β (**A**) Chondrocytes treated with control siRNA or Nrf2 siRNA were analyzed by Western blotting for the expression of nuclear Nrf2, and the representative images were shown. (**B**)The optical density for the Nrf2/Lamin B in chondrocytes was analyzed. Data were shown as mean±SD, *P < 0.05, **P < 0.01, *n* = 6. (**C**–**D**) Chondrocytes treated with 10 ng/mL IL-1β alone, IL-1β combined with PTE or IL-1β combined with PTE and Nrf2 siRNA for 24 h were analyzed by real-time PCR for the expression of COX-2 and iNOS. Data were shown as mean ± 95% CI, **P < 0.01, *n* = 6. (**E**–**F**) Chondrocytes treated with IL-1β alone, IL-1β combined with PTE or IL-1β combined with PTE and Nrf2 siRNA for 24 h were analyzed with nitrite measurement and ELISA for NO and PGE2 expression, respectively. Data were shown as mean ± SD, **P < 0.01, *n* = 6.

RNAi against Nrf2 was utilized to analyze the involvement of Nrf2 in the effect of PTE on IL-1β-induced inflammation. Western blotting showed that the nuclear Nrf2 expression was inhibited significantly in both the control and PTE-treated chondrocytes after Nrf2 siRNA transfection(Figure 2A–2B, *P* < 0.05). As anticipated, IL-1β significantly increased the expression of COX-2 and iNOS mRNA observed by real-time PCR(Figure 2C–2D, *P* < 0.05). IL-1β significantly elevated the release of NO and PGE-2 investigated by nitrite assay and ELISA (Figure 2E–2F, *P* < 0.05). However, PTE could partly attenuate the increase of these inflammatory mediators in the IL-1β-treated chondrocytes (*P* < 0.05), indicating the anti-inflammatory role of PTE. Compared to the cells treated with IL-1β and PTE, Nrf2 inhibition could abolish the inhibitory effect of PTE on the expression of COX-2 and iNOS mRNA and the release of NO and PGE-2 in chondrocytes, suggesting that Nrf2 pathway might mediate the protective role of PTE on chondrocytes under inflammatory condition.

### The involvement of Nrf2 in the anti-ROS production effect of PTE on IL-1β-treated chondrocytes

**Figure 3:**
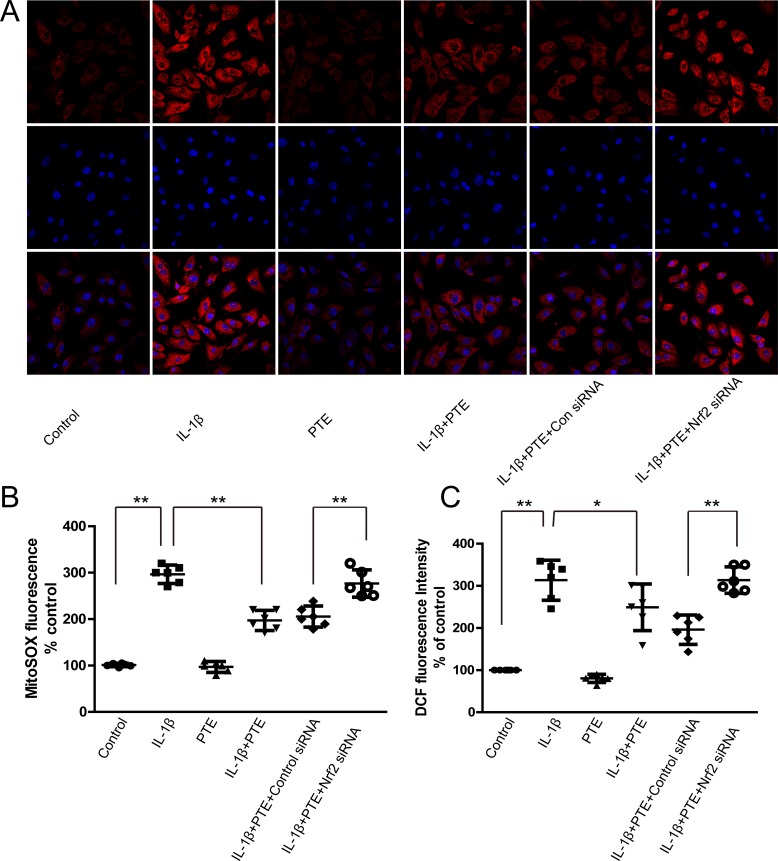
The involvement of Nrf2 in the inhibitory effect of PTE on the ROS production in the chondrocytes treated with IL-1β (**A**) Chondrocytes treated with 10 ng/mL IL-1β alone, IL-1β combined with PTE or IL-1β coupled with PTE and Nrf2 siRNA for 24 h were analyzed for the production of mitochondrial superoxide using MitoSOX Red staining. The representative images were shown. (**B**) The quantitative analysis of MitoSOX Red staining was shown. Data were shown as mean ± 95% CI, **P < 0.01, *n* = 6. (**C**) Chondrocytes treated with IL-1β alone, IL-1β combined with PTE or IL-1β combined with PTE and Nrf2 siRNA for 24 h were analyzed for DCF fluorescence intensity. Data were shown as mean ± 95% CI, *P < 0.05, **P < 0.01, *n* = 6.

The anti-oxidative stress ability of PTE was explored by the MitoSOX-Red and 2,7-dichlorofluorescin diacetate (DCFDA) staining for analyzing the production of mitochondrial superoxide and intracellular ROS. Unsurprisingly, IL-1β markedly enhanced the red fluorescence density of MitoSOX-Red staining, which was partially attenuated by the pre-treatment of PTE, indicating the inhibitory effect of PTE on the generation of mitochondrial superoxide (Figure 3A–3B, *P* < 0.05). Conversely, Nrf2 silencing could reverse the inhibitory role of PTE on the generation of mitochondrial superoxide, indicating that Nrf2 was a potential mediator of the protective effect (Figure 3A–3B). Similar to the result of MitoSOX-Red staining, PTE inhibited the production of total intracellular ROS induced by the IL-1β demonstrated by DCFDA staining (Figure 3C). Also, the Nrf2 siRNA transfection increased the intensity of DCFDA staining, suggesting the involvement of Nrf2 in the effect of PTE on total intracellular ROS production.

### Effect of PTE on osteoarthritis in knee cartilages of SD rats

**Figure 4:**
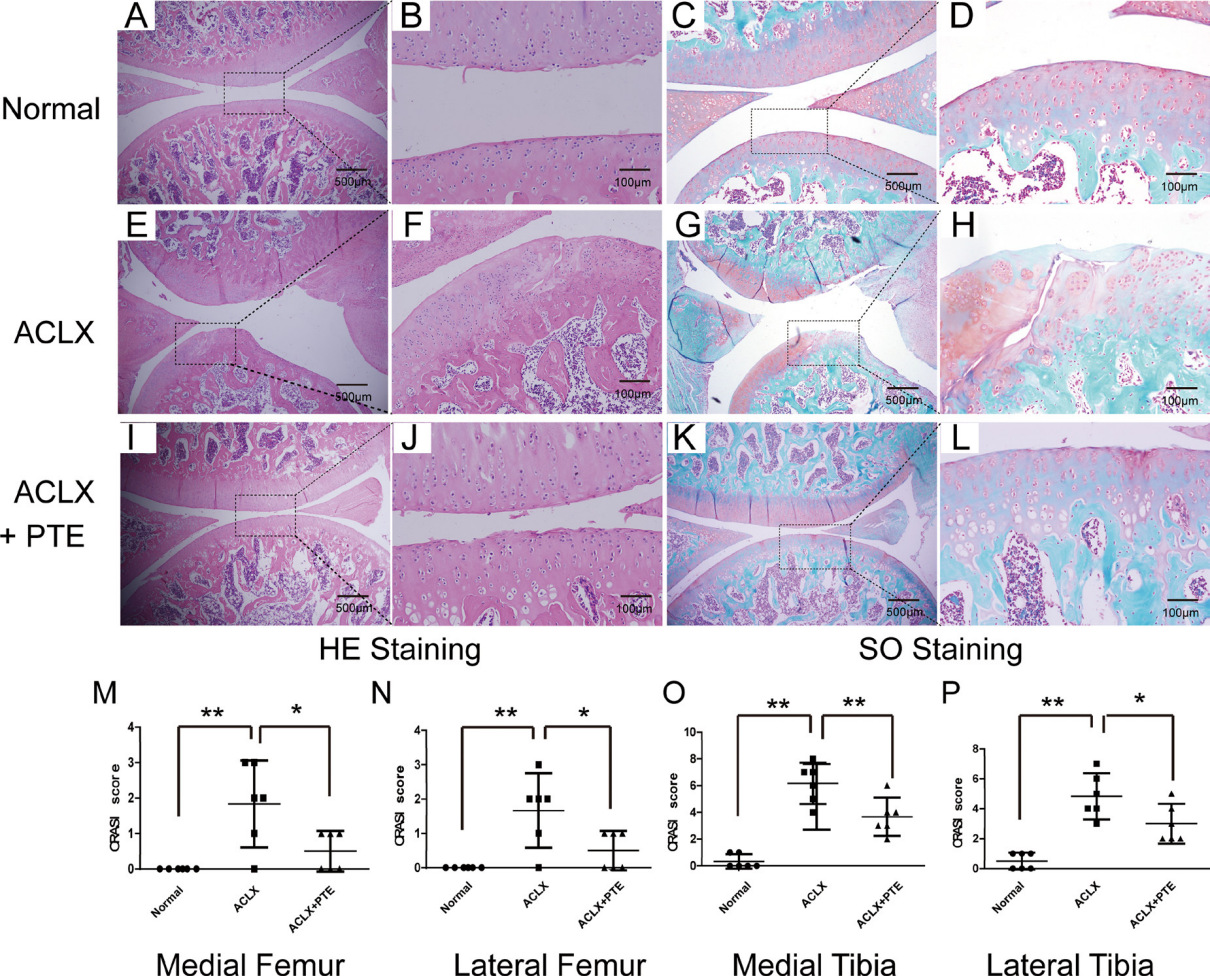
Effect of PTE on knee cartilage degeneration in SD rats (**A**–**D**) The HE and Safranin O/Fast Green staining in the normal right articular cartilage of knee joints. The representative figures were shown. (**E**–**H**) The knee joints of SD rats were treated with anterior cruciate ligament (ACL) transection and partial medial meniscectomy (ACLX). 8 weeks after the surgery, cartilage were stained with HE and Safranin O/Fast Green. (**I**–**L**) The rats with OA were treated with PTE for 8 weeks, and then the knees were also analyzed by HE and Safranin O/Fast Green staining. (**M**–**P**) ORASI score for four quadrants in OA cartilages treated with PTE from histological sections at the same level of joints. Data were shown as mean ± 95% CI, *P < 0.05, **P < 0.01, *n* = 6.

During the surgery and experiment, all the rats were healthy without anesthetic accident, weight loss, and infection. In order to confirm the effect of PTE on the cartilage degeneration and the nuclear-cytoplasmic distribution of Nrf2 protein in cartilages, SD rats with osteoarthritis were treated with PTE for 8 weeks. Compared with the OA cartilage, the PTE-treated OA cartilage showed more smooth, more deposit of proteoglycan in ECM and less formation of fibrillations demonstrated by the HE and Safranin O/Fast Green staining (Figure 4A–4L). Additionally, cell cluster formation and cell density decline could be found in the OA cartilage of ACLX group (Figure 4A–4L). The OARSI score analysis showed that the damage and degeneration of cartilage in medial tibia was most severe, compared with that in medial femur, lateral femur and lateral tibia (Figure 4M–4P). Importantly, PTE could significantly reduced the severity of cartilage degeneration in the four quadrants of OA cartilages (Figure 4M–4P).

### Effect of PTE on Nrf2 expression in OA articular cartilages of SD rats

**Figure 5:**
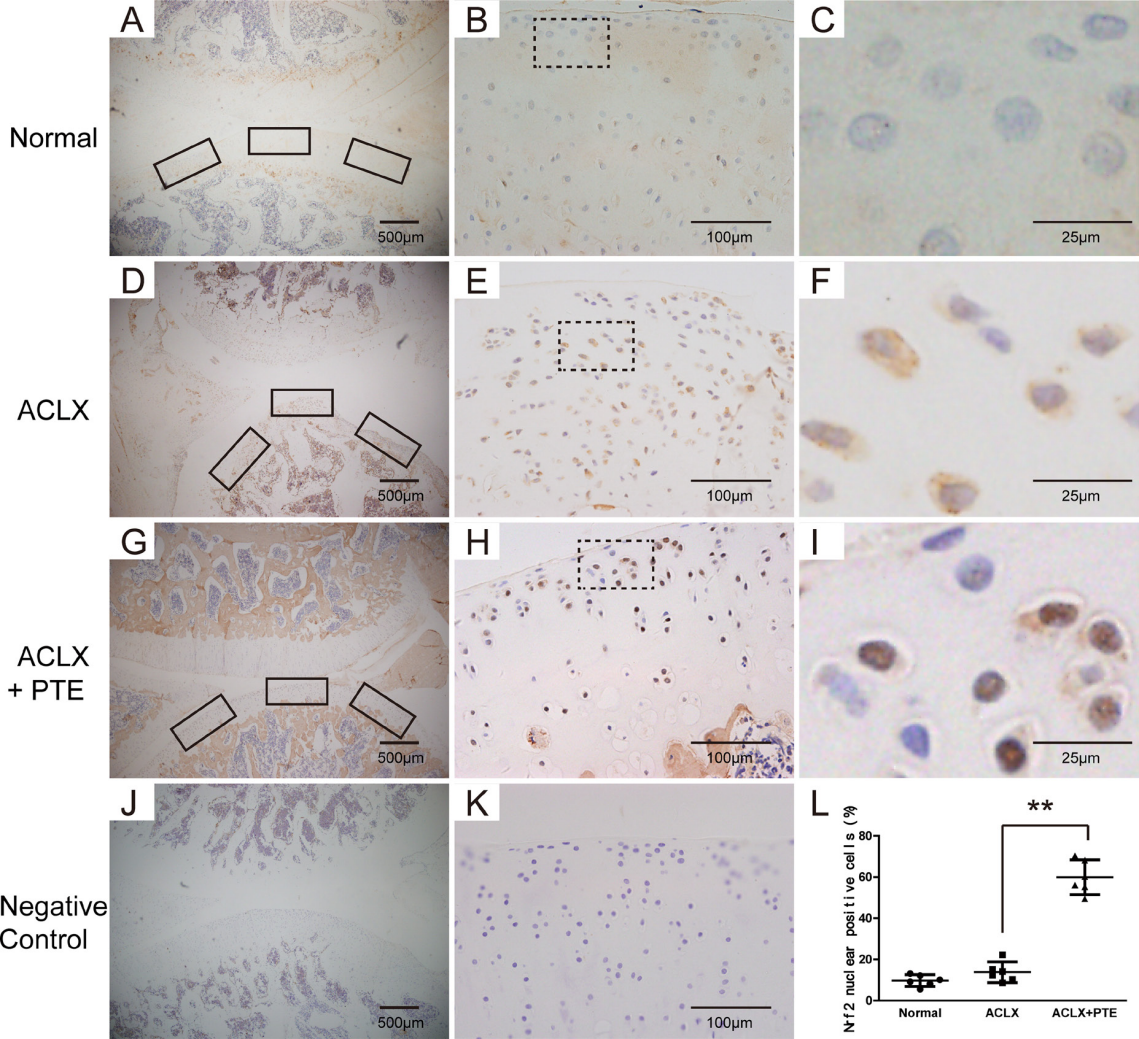
Expression and location of Nrf2 in the knee articular cartilage examined with immunohistochemistry The representative figures were shown. (**A**–**C**) The Nrf2 expression and location in the normal cartilages. The rectangle part in Figure B was enlarged, which is shown in Figure C. (**D**–**F**) The Nrf2 expression and location in the OA cartilages of ACLX group. The rectangle part in figure E was enlarged, which is shown in Figure F. (**G**–**I**) The Nrf2 expression and location in the OA cartilages of ACLX+PTE group. The rectangle part in figure H was enlarged, which is shown in Figure I. (**J**–**K**) The negative control without the primary antibody. (**L**) The percentage of positive cells was calculated by dividing the number of cells with positive Nrf2 in nuclei by the average total cell number in three fields. Data were shown as mean ± 95% CI, **P < 0.01, *n* = 6.

Immunohistochemistry for Nrf2 was performed in the cartilage. As shown in the Figure 5G–5I, the cartilage in the PTE-treated rats exhibited more positive chondrocytes with brown staining in the nuclei, compared with that in the ACLX group, suggesting the stimulatory role of PTE on the nuclear translocation of Nrf2 *in vivo*. In the OA cartilages, the Nrf2 protein was mainly distributed in the cytoplasm (Figure 5D–5F), and the normal cartilage showed less Nrf2 expression in both the cytoplasm and nucleus (Figure 5A–5C). In order to analyze the percentage of Nrf2-positive cells in nuclei, three pictures were taken under 40 magnification in cartilage sections from knees joints, representing the centre, medial and lateral tibial surface. The number of cells with brown staining in the nuclei and the total cell number in each section was counted. Compared with the cartilage in the ACLX group, more percentage of Nrf2-positive cells in nuclei could be found in the PTE-treated cartilage (Figure 5A–5C).

## DISCUSSION

Recently, PTE has been regarded a promising drug to alleviate the cartilage degeneration which is considered as an inflammatory disease. Because the protective role of blueberry on OA has been extensively studied, it is not surprising that PTE could inhibit the inflammation and ROS production in chondrocytes induced by IL-1β. The production of inflammatory mediators including COX-2, iNOS, PGE2, and NO, as well as the mitochondrial and intracellular ROS, was significantly inhibited by PTE. However, Nrf2 silencing could partly abolish the benefits in chondrocytes, suggesting that PTE might inhibit inflammation and ROS production by activating Nrf2 signaling and the following pathways. Finally, we showed that PTE could prevent the cartilage degeneration and promote the nuclear translocation of Nrf2 in the OA articular cartilage, further demonstrating the protective role of PTE on OA *in vivo*. However, the symptomatic recovery in rats has not been described due to the difficult to obtain the data about the symptom.

First, the cytotoxicity of PTE on chondrocytes was checked, and we found that 20 μM PTE with 24 h treatment was not detrimental to the chondrocyte viability. In a previous study, PTE had no cytotoxic effects on the murine macrophages at doses of 1–100 μM with 24 h treatment [24]. However, when the treatment time was extended to 72 h, PTE was found to inhibit the cell viability at a dosage above 30 μM [24].

IL-1β has been regarded as a classic pro-inflammatory and pro-catabolic factor in chondrocytes, contributing to the degradation of ECM [25, 26]. The present study also showed that IL-1β increased the expression of COX-2, PGE2, and iNOS which was an enzyme producing NO. COX-2 and PGE2 are the primary mediators of osteoarthritis pain induced by inflammation [26]. The *in vitro* studies demonstrated that PTE could inhibit the expression of these inflammatory mediators induced by IL-1β, suggesting it as a candidate to relieve the pain symptom of OA. However, PTE could to some extent increase the COX-2 and iNOS expression compared with the control group, suggesting that PTE alone could also activate the low-level inflammation in chondrocytes.

The inflammation and ROS production are initiated in the OA cartilage. Compared with the low level of ROS in normal cartilage, the ROS production was significantly elevated in the OA cartilage [27]. ROS production leads to the imbalance of ECM production, cell senescence, mitochondrial dysfunction, and apoptosis, as well as DNA damage [2]. In the present study, IL-1β increased the production of the total intracellular and mitochondrial ROS, further confirming the induction of mitochondrial dysfunction by IL-1β [10] and suggesting mitochondria as the primary source of ROS production. However, these ROS production induced by IL-1β was inhibited by PTE. Given the contribution of ROS and inflammation to the degradation in cartilage [28], the inhibition of inflammation and ROS production might mediate the protective role of PTE on cartilage degeneration in the rat model of OA.

Although the mechanisms mediating the protective role of PTE included several signaling pathways such as the regulation of Sirt1, extracellular regulated kinase (ERK) and NF-ĸB pathways [29], the activation of Nrf2 pathway was still considered as a promising approach. The nuclear-cytoplasmic distribution of Nrf2 protein altered by the PTE was found *in vitro* and *in vivo*, demonstrating the stimulatory role of PTE on the nuclear translocation. Furthermore, the involvement of Nrf2 in the protective effect of PTE on cartilage degeneration was also proved. As a self-adaptive mechanism in cells, Nrf2 expression was changed in numerous diseases with inflammation and oxidative stress. A significant decrease in nuclear Nrf2 expression was observed in the affected brain regions of Alzheimer disease (AD) and liver of SAMP8 mice with a systemic accumulation of oxidative stress [30, 31]. In the present study, the nuclear Nrf2 protein was not increased in the OA cartilage compared with the normal cartilage, suggesting the inactivation of Nrf2-mediated transcription against oxidative stress and inflammation in OA cartilage.

Under physiological conditions, Nrf2 binds to Kelch-like ECH-associated protein-1 (Keap1), therefore, the dissociation of Nrf2 from Keap1 is the first step of Nrf2 nuclear translocation [32]. Ramkumar et al. [32] found that PTE could disturb the Nrf-Keap1 interaction and promote the nuclear translocation of Nrf2 in the human embryonic kidney cells. Similarly, our finding further validated the finding in chondrocytes. After entering into the nucleus, Nrf2 binds to the ARE and forms the heterodimers with the transcription factors c-Jun and small Maf proteins (G/F/K), stimulating the transcription of antioxidant genes and combating with not only the products of oxidation, but also the oxygen radicals [20].

In conclusion, these results indicate that PTE stimulates the nuclear translocation of Nrf2 and inhibits inflammation and ROS production in the IL-1β-induced chondrocytes. The activation of Nrf2 pathway promotes the transcription of antioxidant genes and inhibits ROS production, as well as the inflammation. PTE could prevent the cartilage degeneration and activate Nrf2 in the OA cartilage.

## MATERIALS AND METHODS

The experimental protocols for animals were approved by Wenzhou Medical University Animal Care and Use Committee (Ethics Reference NO. L-2016-07) (Wenzhou, Zhejiang, China).

### Reagents and antibodies

PTE, hematoxylin, eosin, safranin O, fast green, and type II collagenase were purchased from Sigma-Aldrich (St. Louis, MO, USA). The primary antibodies for Nrf2, β-actin, and Lamin B and ELISA kits for Prostaglandin E2 (PGE2) were acquired from Abcam (Cambridge, UK). The secondary antibodies for immunohistochemistry and Western blotting, DAPI, cytoplasmic and nuclear protein extraction reagents, DCFDA assay kits were from Beyotime (Shanghai, China). The cell culture reagents including Dulbecco modified Eagle medium (DMEM), fetal bovine serum (FBS), and 0.25% trypsin were bought from Gibco (Grand Island, NY, USA). Cell Counting Kit-8 (CCK-8) was obtained from Dojindo (Tokyo, Japan). Real-time PCR reagents including TRIzol and SYBR Premix Ex Taq mixture were procured from Takara (Takara Bio, Otsu, Japan). The cDNA synthesis kit was purchased from MBI Fermentas (St Leon-Rot, Germany). MitoSOX Red staining kit was obtained from Molecular Probes (Invitrogen, Carlsbad, CA, USA).

### Rat chondrocyte culture

The chondrocyte extraction and culture were carried out according to the previous reports [33]. 5 male 250–300 g SD rats (2.5 months old) were euthanized by CO2 inhalation. The bilateral cartilages of knee joints were totally resected, followed by cutting with Micro scissors, digestion with 0.25% Trypsin-EDTA for 30 min and collagenase II for 4 h. The cell aggregates after digestion and washing were filtrated through 150-μM mesh, then high-glucose (4.5 g/L) DMEM with 10% FBS and 1% penicillin/streptomycin were used to culture chondrocytes in an incubator with 5% CO_2_ at 37°C. In order to eliminate the influence of de-differentiation in chondrocytes, the second passage cells were used in the downstream experiments.

### Experimental design

Primary rat chondrocytes were isolated six times, and the following experiments were repeated six times using the isolated chondrocytes each time. First, the cellular toxicity of PTE was examined by CCK-8 assay. Then the effect of PTE on Nrf2 activation was explored, followed by the investigation of its protective role against inflammatory and ROS production, as well as the involvement of Nrf2. 10 ng/mL IL-1β was used to promote inflammation and ROS production in the chondrocytes. PTE were added to the medium 2 h prior to the IL-1β. RNAi was used for the silence of Nrf2. 48 h post transfection, the PTE and IL-1β were added successively.

### Cell counting kit-8 assay

Different concentrations of PTE (0–80 μM) for 24 h and 20 μM PTE with 0–48 h treatments were used to treat the 2 × 10^3^ chondrocytes in a 96-well plate. 10 μL reagent was then added to the medium for 1 h at 37°C. The absorbance was measured at 450 nm in the microplate reader (FlexStation, Molecular Devices).

### Immunofluorescence

2 × 10^4^ chondrocytes cultured in 48-well plate were treated with 10 and 20 μM PTE for 24 h. The chondrocytes were fixed with 4% paraformaldehyde for 10 min at 4°C. Subsequently, the cellular membrane was permeabilized with 0.2%Triton X-100 for 15 min and blocked with 5% BSA for 30 min. The chondrocytes were incubated with the Nrf2 primary antibody (1:100) at 4°C for 12–16 h, followed by incubation with FITC conjugated secondary antibodies. The nuclei were counterstained with DAPI. Finally, the chondrocytes were observed under a confocal microscope (Leica TCS SP8,Germany).

### Western blotting

The nuclear and cytoplasmic protein from the chondrocytes was isolated as recommended by the manufacturer using the extraction kits (Beyotime, Shanghai, China). The nuclear and cytoplasmic protein concentration was determined using an Enhanced BCA Protein Assay Kit. The total protein was resolved on sodium dodecyl sulfate-polyacrylamide gel electrophoresis (SDS-PAGE) and transferred onto the polyvinylidene difluoride (PVDF) membrane (BIO-RAD USA). After blocking with 5% nonfat milk for 2 h, the membrane was incubated with the Nrf2 primary antibody (1:1000) and HRP-conjugated secondary antibodies. ECL Plus reagent was used to expose the membrane in an enhanced chemiluminescence detection system (PerkinElmer, USA). Semi-quantitative analysis of band intensity was analyzed byAlphaEaseFC 4.0 software.

### Nrf2 siRNA transfection

The sequences of small interfering RNAs (siRNA), shown in Table 1 were designed as previously described [33, 34] and synthesized by GenePharma (Shanghai, China). Cells were cultured in a 6-well tissue culture plate (2 × 10^5^ cells/well). siRNA was delivered into chondrocytes using Lipofectamine 2000 (Invitrogen) following the manufacturer's instructions. 48 h post-transfection, the infected cells were treated as described in the experimental design. The non-specific non-targeting siRNA (scrambled) was used as a control. Western blotting consequently examined the inhibitory efficiency of specific silencing using the nuclear extracts.

**Table 1:** The sequences of small interfering RNAs (siRNAs)

Primer	Direction	Sequence 5′→3′
*Nrf2*	Sense	GGCAUUUCACUGAACACAA dTdT
	Antisense	UUGUGUUCAGUGAAAUGCCdGdG
Negative control	Sense	UUCUCCGAACGUGUCACGUdT
	Antisense	ACGUGACACGUUCGGAGAAdTdT

### Real-time PCR

After treatments as the experimental design described, the total RNA of chondrocytes was extracted using TRIzol reagent, followed by the synthesis of cNDA using 1μg total RNA. The RNA was quantified with a spectrophotometer (NanoDrop ND-1000; Thermo Scientific, Wilmington, DE) by controlling the range of ratio OD 260/280 between 1.8 and 2.0. The RT-PCR 20 μL reaction included 2 μL of 2-fold diluted cDNA, 10 μL of 2 × SYBR Premix Ex Taq mixture, 0.2 μmol/L of each primer and sterile distilled water. The 8-strip PCR tubes and placed in a LightCycler (Roche, Mannheim, Germany) for detection. The thermocycler parameters were 50°C for 2 min, 95°C for 30s, 40 cycles at 95°C for 5 s, and 60°C for 34 s [35]. DNA contamination was evaluated using a non-template control. The primers of COX-2, iNOS, and GAPDH of rats were shown in Table 2. After the reaction, the cycle threshold (Ct) values were obtained and normalized against the housekeeping gene GAPDH. The relative mRNA levels of each target gene were calculated by the 2^−ΔΔCt^ method.

**Table 2:** Primer sequences for COX-2, iNOS and GAPDH

Gene		sequences (5′–3′)	Accession no.
COX-2	forward	GGTGAAAACTGTACTACGCCGA	S67722
COX-2		ACTCCCTTGAAGTGGGTCAG	
iNOS	forward	GAAACTTCTCAGCCACCTTGG	AB250951
iNOS		CCGTGGGGCTTGTAGTTGAC	
GAPDH	forward	TGATTCTACCCACGGCAAGT	M17701
GAPDH		AGCATCACCCCATTTGATGT	

### Nitrite measurement

The nitrite level in the supernatant of the medium was assessed by the classic Griess method [36]. After treatments, Griess reagents I and II were added to the supernatant to the 96-well plates and incubated at 37°C for 3 min. Absorbance was measured with the microplate reader at 540 nm, and the nitrite concentration calculated from the standard curve.

### ELISA assay

After treatment with PTE and IL-1β for 24 h, the culture supernatants were obtained. The concentration of PGE-2 was measured by ELISA according to the manufacturer's instructions. The sensitivity of PGE-2 ELISA assay was 13.4pg/mL.

### MitoSOX red staining for mitochondrial ROS

MitoSOX Red is a novel dye targeting the mitochondria and emitting red fluorescence when oxidized by the mitochondrial Superoxide Anion (O^2^-). First, 50μg MitoSOX dye dissolved in 13 μL DMSO was added into a serum-free medium for a 5 mM stock concentration. 5 × 10^4^ chondrocytes cultured in glass dishes were incubated with the 1 mL 5 μM working solution for 15 min at 37°C, followed by DAPI staining for 5 min at 37°C. After washing with PBS three times, the cells cultured in fresh DMEM were observed under the confocal microscope (Leica TCS SP8,Germany).

### DCFDA assay for intracellular ROS

DCFDA assay is a classic method for analyzing the intracellular level of hydroxyl, peroxyl, and other ROS, performed according to a previous report [37]. After treatments as described in the experimental design, chondrocytes in 96-well plates were incubated with 10 μM DCFDA in the serum-free medium at 37°C for 20 min. Subsequently, chondrocyte suspensions were obtained by harvesting with 0.25% trypsin-EDTA and centrifugation. ROS level was immediately assessed using a flow cytometry (FACSCaliber; Becton Dickinson, Heidelberg, Germany) to detect the mean fluorescence intensity (MFI) with an excitation wave length of 488 nm and an emission wave length of 525 nm.

### Establishment and treatment of an OA model of knee joints in SD rats

The osteoarthritis Sprague-Dawley (SD) rat model was established as previously described [38]. Briefly, 18 male 200–300 g SD (2 months old) rats in the conventional housing were anesthetized by the10% (w/v) chloral hydrate (0.2 mL/100 g) by intraperitoneal injection and randomly divided into 3 groups including normal, anterior cruciate ligament (ACL) transection and partial medial meniscectomy (ACLX), and ACLX+PTE groups. The surgeries were performed on the right knee joints. After exposing the anterior cruciate ligament (ACL) and medial meniscus via the medial approach, the ACL was transected, and the partial meniscus was resected (ACLX). The rats in the ACLX, and ACLX+PTE groups received the above surgery. The rats in the normal group underwent the same incision and exposure without the resection of ACL and meniscus. Subsequent to the surgery, all the animals were administered solvent agent-sunflower oil, and the rats in the ACLX+PTE group received the 30 mg/kg PTE, daily, p.o. in sunflower oil for 8 weeks. 8 weeks after the surgery, 6 rats in every group were euthanized for the histological analysis and immunohistochemistry.

### Histological assessment

The whole knee joints of each rat were fixed in 10% neutral formalin and decalcified with10% EDTA for 2 months, and then paraffin-embedded for midsagittal serial sectioning. The sections were HE stained to assess the destruction of cartilage. In order to investigate the matrix proteoglycans expression, Safranin O/Fast Green staining was performed on the sections. Cartilage degradation was quantified using the Osteoarthritis Research Society International (OARSI) cartilage grading system [39]. Three independent and blinded observers who were not aware of the treatment procedures received by each joint scored each section, and the scores for all of the sections cut from the medial femur, lateral femur, medial tibia and lateral tibia in 6 rats were averaged respectively. In each quadrant, at least 3 sections were scored, and the scores were averaged within groups. The higher score indicates the more cartilage degeneration.

### Immunohistochemistry

The above sections were used to perform the immunohistochemistry. After deparaffinization and graded rehydration, the endogenous peroxidase activity was blocked with 3% H_2_O_2_ for 10 min. The antigen was retrieved by the conventional heat method, followed by blocking with 5% BSA and permeabilization with 1% Tween-20 in PBS for 30 min. The sections were incubated with the primary antibodies against Nrf2 (1:200) and PBS as negative controls for 12–16 h at 4°C, followed by HRP-conjugated secondary antibodies and counterstained with hematoxylin. Finally, the sections were microscopically examined. To assure the accuracy, three sections from the 6 knee joints were utilized to observe the expression of Nrf2 in the articular cartilage. In order to analyze the percentage of cells with positive Nrf2 in nuclei, three pictures were taken under 40× magnification in cartilage sections from knees joints, representing the centre, medial and lateral tibial surface. The number of cells with brown staining in the nuclei and the total cell number in each section was counted.

### Statistical analysis

The differences between the groups were evaluated by ANOVA using the SPSS15 package (SPSS Inc., Chicago, IL, USA). If the result of ANOVA had significance, the Bonferroni test was used to explore the differences between the two groups. *P* < 0.05 was statistically significant.

## Abbreviations

PTE: pterostilbene
Nrf2: Nuclear factor erythroid 2-related factor 2
SD: Sprague-Dawley
OA: osteoarthritis
ECM: extracellular matrix
TNF-α: tumor necrosis factor-alpha
IL-1β: interleukin-1β
COX-2: cyclooxygenase 2
NO: nitric oxide
PGE2: prostaglandin E2
MMPs: matrix metalloproteinase
ROS: reactive oxygen species
NSAIDs: non-steroidal anti-inflammatory drugs
ARE: antioxidant response element
HO1: heme oxygenase 1
NQO1: AD(P)H-quinone oxidoreductase-1
GPx: glutathione peroxidase
γ-GCS: γ-glutamylcysteine synthetase
CAT: catalase
SOD: superoxide dismutase

## References

[1] Agaliotis M, Fransen M, Bridgett L, Nairn L, Votrubec M, Jan S, Heard R, Mackey M (2013). Risk factors associated with reduced work productivity among people with chronic knee pain. Osteoarthritis Cartilage.

[2] Lepetsos P, Papavassiliou AG (2016). ROS/oxidative stress signaling in osteoarthritis. Biochim Biophys Acta.

[3] Bijlsma JW, Berenbaum F, Lafeber FP (2011). Osteoarthritis: an update with relevance for clinical practice. Lancet.

[4] Mobasheri A, Platt N, Thorpe C, Shakibaei M (2006). Regulation of 2-deoxy-D-glucose transport, lactate metabolism, and MMP-2 secretion by the hypoxia mimetic cobalt chloride in articular chondrocytes. Ann N Y Acad Sci.

[5] Rahmati M, Mobasheri A, Mozafari M (2016). Inflammatory mediators in osteoarthritis: A critical review of the state-of-the-art, current prospects, and future challenges. Bone.

[6] Bondeson J, Blom AB, Wainwright S, Hughes C, Caterson B, van den Berg WB (2010). The role of synovial macrophages and macrophage-produced mediators in driving inflammatory and destructive responses in osteoarthritis. Arthritis Rheum.

[7] Legendre F, Dudhia J, Pujol JP, Bogdanowicz P (2003). JAK/STAT but not ERK1/ERK2 pathway mediates interleukin (IL)-6/soluble IL-6R down-regulation of Type II collagen, aggrecan core, and link protein transcription in articular chondrocytes. Association with a down-regulation of SOX9 expression. J Biol Chem.

[8] Yin W, Park JI, Loeser RF (2009). Oxidative stress inhibits insulin-like growth factor-I induction of chondrocyte proteoglycan synthesis through differential regulation of phosphatidylinositol 3-Kinase-Akt and MEK-ERK MAPK signaling pathways. J Biol Chem.

[9] Yu SM, Kim SJ (2015). The thymoquinone-induced production of reactive oxygen species promotes dedifferentiation through the ERK pathway and inflammation through the p38 and PI3K pathways in rabbit articular chondrocytes. Int J Mol Med.

[10] Yasuhara R, Miyamoto Y, Akaike T, Akuta T, Nakamura M, Takami M, Morimura N, Yasu K, Kamijo R (2005). Interleukin-1beta induces death in chondrocyte-like ATDC5 cells through mitochondrial dysfunction and energy depletion in a reactive nitrogen and oxygen species-dependent manner. Biochem J.

[11] Richette P, Latourte A, Frazier A (2015). Safety and efficacy of paracetamol and NSAIDs in osteoarthritis: which drug to recommend?. Expert Opin Drug Saf.

[12] Estrela JM, Ortega A, Mena S, Rodriguez ML, Asensi M (2013). Pterostilbene: Biomedical applications. Crit Rev Clin Lab Sci.

[13] Roupe KA, Remsberg CM, Yanez JA, Davies NM (2006). Pharmacometrics of stilbenes: seguing towards the clinic. Curr Clin Pharmacol.

[14] Remsberg CM, Yanez JA, Ohgami Y, Vega-Villa KR, Rimando AM, Davies NM (2008). Pharmacometrics of pterostilbene: preclinical pharmacokinetics and metabolism, anticancer, antiinflammatory, antioxidant and analgesic activity. Phytother Res.

[15] Li J, Ruzhi D, Hua X, Zhang L, Lu F, Coursey TG, Pflugfelder SC, Li DQ (2016). Blueberry Component Pterostilbene Protects Corneal Epithelial Cells from Inflammation via Anti-oxidative Pathway. Sci Rep.

[16] Zhang L, Zhou G, Song W, Tan X, Guo Y, Zhou B, Jing H, Zhao S, Chen L (2012). Pterostilbene protects vascular endothelial cells against oxidized low-density lipoprotein-induced apoptosis in vitro and in vivo. Apoptosis.

[17] Chiou YS, Tsai ML, Nagabhushanam K, Wang YJ, Wu CH, Ho CT, Pan MH (2011). Pterostilbene is more potent than resveratrol in preventing azoxymethane (AOM)-induced colon tumorigenesis via activation of the NF-E2-related factor 2 (Nrf2)-mediated antioxidant signaling pathway. J Agric Food Chem.

[18] Bhakkiyalakshmi E, Sireesh D, Sakthivadivel M, Sivasubramanian S, Gunasekaran P, Ramkumar KM (2016). Anti-hyperlipidemic and anti-peroxidative role of pterostilbene via Nrf2 signaling in experimental diabetes. Eur J Pharmacol.

[19] Zhang DD (2006). Mechanistic studies of the Nrf2-Keap1 signaling pathway. Drug Metab Rev.

[20] Muller T, Hengstermann A (2012). Nrf2: friend and foe in preventing cigarette smoking-dependent lung disease. Chem Res Toxicol.

[21] Xue F, Huang JW, Ding PY, Zang HG, Kou ZJ, Li T, Fan J, Peng ZW, Yan WJ (2016). Nrf2/antioxidant defense pathway is involved in the neuroprotective effects of Sirt1 against focal cerebral ischemia in rats after hyperbaric oxygen preconditioning. Behav Brain Res.

[22] Shimizu Y, Nicholson CK, Lambert JP, Barr LA, Kuek N, Herszenhaut D, Tan L, Murohara T, Hansen JM, Husain A, Naqvi N, Calvert JW (2016). Sodium Sulfide Attenuates Ischemic-Induced Heart Failure by Enhancing Proteasomal Function in an Nrf2-Dependent Manner. Circ Heart Fail.

[23] Bhakkiyalakshmi E, Shalini D, Sekar TV, Rajaguru P, Paulmurugan R, Ramkumar KM (2014). Therapeutic potential of pterostilbene against pancreatic beta-cell apoptosis mediated through Nrf2. Br J Pharmacol.

[24] Nikhil K, Sharan S, Roy P (2015). A pterostilbene derivative suppresses osteoclastogenesis by regulating RANKL-mediated NFkappaB and MAPK signaling in RAW264.7 cells. Pharmacol Rep.

[25] Yang Y, Wang Y, Wang Y, Zhao M, Jia H, Li B, Xing D (2016). Tormentic Acid Inhibits IL-1beta-Induced Inflammatory Response in Human Osteoarthritic Chondrocytes. Inflammation.

[26] Wang SN, Xie GP, Qin CH, Chen YR, Zhang KR, Li X, Wu Q, Dong WQ, Yang J, Yu B (2015). Aucubin prevents interleukin-1 beta induced inflammation and cartilage matrix degradation via inhibition of NF-kappaB signaling pathway in rat articular chondrocytes. Int Immunopharmacol.

[27] Altay MA, Erturk C, Bilge A, Yapti M, Levent A, Aksoy N (2015). Evaluation of prolidase activity and oxidative status in patients with knee osteoarthritis: relationships with radiographic severity and clinical parameters. Rheumatol Int.

[28] Berenbaum F, Griffin TM, Liu-Bryan R (2017). Review: Metabolic Regulation of Inflammation in Osteoarthritis. Arthritis Rheumatol.

[29] McCormack D, McFadden D (2013). A review of pterostilbene antioxidant activity and disease modification. Oxid Med Cell Longev.

[30] Tomobe K, Shinozuka T, Kuroiwa M, Nomura Y (2012). Age-related changes of Nrf2 and phosphorylated GSK-3beta in a mouse model of accelerated aging (SAMP8). Arch Gerontol Geriatr.

[31] Ramsey CP, Glass CA, Montgomery MB, Lindl KA, Ritson GP, Chia LA, Hamilton RL, Chu CT, Jordan-Sciutto KL (2007). Expression of Nrf2 in neurodegenerative diseases. J Neuropathol Exp Neurol.

[32] Ramkumar KM, Sekar TV, Foygel K, Elango B, Paulmurugan R (2013). Reporter protein complementation imaging assay to screen and study Nrf2 activators in cells and living animals. Anal Chem.

[33] Jiang LB, Lee S, Wang Y, Xu QT, Meng DH, Zhang J (2016). Adipose-derived stem cells induce autophagic activation and inhibit catabolic response to pro-inflammatory cytokines in rat chondrocytes. Osteoarthritis Cartilage.

[34] Cortese MM, Suschek CV, Wetzel W, Kroncke KD, Kolb-Bachofen V (2008). Zinc protects endothelial cells from hydrogen peroxide via Nrf2-dependent stimulation of glutathione biosynthesis. Free Radic Biol Med.

[35] Yin XF, Jiang LB, Ma YQ, Xu J, Gu HJ, Wu XH, Li XL, Dong J (2015). Decreased Zn(2+) Influx Underlies the Protective Role of Hypoxia in Rat Nucleus Pulposus Cells. Biol Trace Elem Res.

[36] Kissner R, Koppenol WH (2005). Qualitative and quantitative determination of nitrite and nitrate with ion chromatography. Methods Enzymol.

[37] Deng R, Hua X, Li J, Chi W, Zhang Z, Lu F, Zhang L, Pflugfelder SC, Li DQ (2015). Oxidative stress markers induced by hyperosmolarity in primary human corneal epithelial cells. PLoS One.

[38] Chu X, You H, Yuan X, Zhao W, Li W, Guo X (2013). Protective effect of lentivirus-mediated siRNA targeting ADAMTS-5 on cartilage degradation in a rat model of osteoarthritis. Int J Mol Med.

[39] Gerwin N, Bendele AM, Glasson S, Carlson CS (2010). The OARSI histopathology initiative - recommendations for histological assessments of osteoarthritis in the rat. Osteoarthritis Cartilage.

